# Bone biomarkers in post-polio clinic patients

**DOI:** 10.3389/fendo.2025.1568981

**Published:** 2025-06-06

**Authors:** Seyed Pezhman Madani, Richard Kremer, Ami Grunbaum, Shaddam Bagmar, Andrea Benedetti, Daria A. Trojan

**Affiliations:** ^1^ Department of Neurology, Montreal Neurological Institute-Hospital, McGill University Health Centre, McGill University, Montreal, QC, Canada; ^2^ Division of Endocrinology, Department of Medicine, Royal Victoria Hospital, McGill University Health Centre, Montreal, QC, Canada; ^3^ Bone and Mineral Unit, Royal Victoria Hospital, McGill University Health Centre, Montreal, QC, Canada; ^4^ Division of Biochemistry, Department of Medicine, Royal Victoria Hospital, McGill University Health Centre, Montreal, QC, Canada; ^5^ Department of Epidemiology, Biostatistics, and Occupational Health, McGill University, Montreal, QC, Canada

**Keywords:** osteoporosis, poliomyelitis, osteocalcin, C-telopeptide, bisphosphonates

## Abstract

**Introduction:**

Osteoporosis is common in post-polio clinic patients, and is reported in 30%to 50% of middle-aged individuals with previous polio. The levels of bone biomarkers (calcium regulating hormones, bone metabolism markers, and bone turnover markers), and the response of bone turnover markers to bisphosphonates is unknown in post-polio patients with osteoporosis.

**Objectives:**

1) To describe serum levels of bone biomarkers in post-polio clinic patients with osteoporosis and compare these levels to those in controls with osteoporosis without neurological disease. 2) To examine the change in serum levels of bone biomarkers in post-polio patients following at least six months of treatment with bisphosphonates and compare these changes to controls.

**Methods:**

We conducted a retrospective chart review of Post-Polio and Bone Metabolism Clinic charts of our center. Patients without osteoporosis, and incomplete lab data were excluded. For the second objective, patients untreated with bisphosphonates were excluded.

**Results:**

Mean age and proportion of females were similar in post-polio patients (n=25) and controls (n=31) (66.3 ± 8.1 vs 66.2 ± 10.9 years, 52% vs 61%). Mean baseline serum levels of calcium, calcium regulating hormones [parathyroid hormone (PTH), 25-hydroxy Vitamin D), and serum bone turnover makers (sBTM’s; osteocalcin, C-telopeptide, non-specific alkaline phosphatase (ALP)] were normal. PTH (4.4 ± 1.7 vs 5.5 ± 2.3 pmol/L, p=0.05), ALP (63.9 ± 15.8 vs 76.2 ± 26.7 U/L, p=0.04), osteocalcin (18.3 ± 8.8 vs 26.9 ± 8.4 ng/ml, p<0.01), and C-telopeptide (0.35 ± 0.2 vs 0.55 ± 0.21 microgram/L, p=0.01) were significantly lower in post-polio patients. After ≥ six months of treatment with bisphosphonates, sBTM’s declined significantly in both groups, with a significantly greater reduction in controls for osteocalcin (p<0.01) and C-telopeptide (p=0.02).

**Conclusions:**

While mean levels of all evaluated bone biomarkers were normal, PTH and sBTMs were significantly lower in post-polio patients with osteoporosis compared to controls, indicating reduced bone turnover. With bisphosphonate treatment, osteocalcin and C-telopeptide declined significantly in both groups, but significantly more in controls than in post-polio patients. These results indicate that BTM’s could be useful for monitoring treatment response in post-polio patients.

## 1 Introduction

Acute paralytic poliomyelitis can produce permanent flaccid weakness and muscular atrophy, with reduced mobility, and an increased risk for falls (1). There are 15 to 20 million survivors of paralytic poliomyelitis worldwide. Twenty to 75% of these individuals can also develop post-poliomyelitis syndrome (PPS) with further weakness later in life (2). Osteoporosis is common in post-polio patients (3), is observed in approximately 30-50% of middle-aged individuals with previous polio (4, 5), and most commonly involves the hip region, especially in the weaker lower extremity (6). In addition to the known risk factors for osteoporosis in the general population (7), the close interaction of bone and skeletal muscle mass and the post-polio patients’ baseline mobility reduction and weakness (8, 9) predispose them to a higher risk of osteoporosis and its complications. Recurrent falls in approximately 40% of these patients (10), and at least one bone fracture in 35% to more than half, produce a further deterioration in their mobility and physical activity (11). Most post-polio patients requiring operative treatment for a femoral fracture do not regain their previous ambulatory capacity (12). Accordingly, a multidisciplinary approach is suggested for prevention, appropriate diagnosis, and treatment of osteoporosis in these patients.

BMD is the standard test to detect osteoporosis in normal and paretic patients (13, 14). Bisphosphonates, such as alendronate, risedronate, and zoledronic acid, are the most widely used first-line medications to treat osteoporosis in the general population (15, 16). Bisphosphonates have also been shown to improve the bone mineral density (BMD) of patients with Duchenne muscular dystrophy in a non-randomized, controlled trial (17), and in post-polio patients in a retrospective study completed by us (18).

An extensive array of bone biomarkers is used in clinical practice and research. These include markers of bone turnover [for example, formation markers osteocalcin (OC), type 1 procollagen amino terminal peptides (PINP and bone specific alkaline phosphatase (ALP), and resorption marker carboxy-terminal cross-linked telopeptide of type 1 collagen (CTX)] and bone metabolism [for example, calcium, phosphate and the hormones parathyroid hormone (PTH) and 25 hydroxy vitamin D (25D)]. Bone metabolism markers and non-specific ALP are not suggested as sensitive tools for evaluating the risk of bone fractures (19, 20). However, serum bone turnover biomarkers (sBTMs) have been recommended as rapid, available, cost-effective, and valuable indicators of the response to treatment and risk of bone fractures (21–24) in osteoporotic patients and in the evaluation and management of post-menopausal osteoporosis (25).

Osteocalcin, also known as bone gamma-carboxy glutamic acid-containing protein, is a marker of bone formation produced by osteoblasts, is positively associated with increased BMD during treatment and is suggested as a valuable tool for the clinical assessment of osteoporosis treatment (26). CTX, released during collagen degradation, has been suggested as a specific and sensitive biomarker of bone resorption (26). It is a valuable indicator of rapid response to treatment in osteoporotic postmenopausal women (27). Even though sBTMs in addition to BMD are useful in monitoring response to osteoporosis treatment and identifying high-risk patients (24, 28, 29), there are no published reports describing sBTM changes in response to osteoporosis treatment in patients with previous paralytic polio. The present study will build on our previous work in the area of osteoporosis in post-polio patients (6, 18) and is aimed to evaluate serum levels of hormones regulating calcium homeostasis, bone metabolism biomarkers, and sBTMs in osteoporotic post-polio patients and how they respond to bisphosphonate treatment.

The specific objectives of this study were to (1) describe serum levels of calcium regulating hormones (PTH, 25D), bone metabolism markers (calcium), and bone turnover markers (OC, CTX and non-specific ALP) in post-polio patients with osteoporosis before treatment, and compare these levels to those in controls with osteoporosis without neurological disease (2); examine the change in serum levels of bone biomarkers following at least six months of treatment with bisphosphonates and compare these changes to those found in controls with osteoporosis without neurological disease; and (3) evaluate the relationship between baseline 25D levels and changes in bone biomarkers with bisphosphonate treatment for at least six months.

## 2 Materials and methods

### 2.1 Study design

We conducted a retrospective chart review study. Data were obtained from a chart review of patients who had been evaluated and followed at Post-Polio and Bone Metabolism Clinics of the McGill University Health Centre (MUHC). Our institutional research ethics board approved the study.

### 2.2 Study population

Post-polio patient charts were reviewed between September 2018 and July 2020. For objective 1, all post-polio patients with osteoporosis who had or had not been started on bisphosphonates and who had serum levels of bone metabolism markers measured were included. Bone biomarkers are requested for all patients evaluated in the Bone Metabolism Clinic. For objective 2, all post-polio patients with osteoporosis who were naïve to prior treatment, had been on bisphosphonates for at least six months, and had sBTM levels measured before and after six months of treatment with bisphosphonates were included. A treatment time of at least six months was chosen since for most patients sBTM’s reach a nadir in three to six months (27). Exclusion criteria were (1) absence of history and physical examination consistent with previous paralytic polio, or osteoporosis (2); presence of other medical problems which can cause osteoporosis (e.g. untreated thyroid disease, Paget’s disease, primary hyperparathyroidism, Cushing’s syndrome, gastrectomy, malabsorption syndrome, pituitary adenoma) (3); estimated glomerular filtration rate (eGFR) of <60 mL/min since renal function can influence bone marker clearance (4); current use of medications, which can cause osteoporosis (e.g. steroids, certain anticonvulsants) (5); presence of significant neurologic difficulties (other than paralytic polio and PPS (6); incomplete lab data.

Control subjects were matched to the post-polio patients for age and sex from a chart review of non-polio patients attending the MUHC Bone Metabolism Clinic. Inclusion/exclusion criteria for controls were similar to those for post-polio patients, except that the control subjects did not have histories and physical exams consistent with previous polio.

### 2.3 Data collection and outcome measures

The dependent variables were serum levels of hormones regulating calcium homeostasis (PTH, 25D), bone metabolism markers (calcium), and sBTMs (OC, CTX, and non-specific ALP), all assessed primarily at the MUHC central laboratory for all measurements in both groups. The same assays were used. In the MUHC laboratory, PTH and 25D were measured on Beckman DXI platforms via paramagnetic particle chemiluminescent two site immunoenzymatic (CMIA, sandwich) assay and two-step competitive binding immunoenzymatic assay, respectively. Calcium and ALP were assessed on Beckman DXC platforms via indirect potentiometry and enzymatic kinetic rate assay, respectively. OC and CTX were measured on a Roche Cobas E411 platform via electrochemiluminescence (ECLIA).

A standardized form was used to collect clinical data on Post-Polio Clinic patients. Data on the following independent variables were obtained: BMD at the hip and lumbar spine, T-scores at the femoral neck and lumbar spine, dates of BMD assessments, date of birth, height, weight, sex, ethnicity, age at polio, severity of polio, medications used, presence of other medical difficulties, surgical history, menopausal status, history of fractures, dates of fractures, smoking history, alcohol abuse history, serum testosterone level in men, mobility aids used (cane, crutch, orthosis, wheelchair), type of bisphosphonate introduced, date when bisphosphonate was started, supplements of calcium and vitamin D with dosages, the reason for discontinuation of bisphosphonate, and date of discontinuation of bisphosphonate. Data on some of these variables was gathered to correct our comparisons between study groups for factors that could contribute to osteoporosis and levels of bone biomarkers. Data on bone density assessed primarily at the MUHC at the femoral neck and lumbar spine (g/cm2) were obtained. According to the World Health Organization (WHO) criteria, osteoporosis was defined as a T score at or below -2.5 (30). Bone density of the hip had been routinely assessed on the left in the control group, but some post-polio patients had been assessed bilaterally. Severity of polio at acute polio and at time of BMD was measured on a 0 to 6 scale with 6 being most severe (0, no weakness; 0.5, partial weakness; 1, complete paralysis for each of limbs; respiratory impairment: 0, no impairment; 0.5, partial impairment; 1, use of iron lung or respirator; speech/swallowing impairment: 0, no impairment; 0.5, partial impairment; 1 severe difficulties) A similar measuring scheme has previously been validated (31).

Coded patients’ data were entered into a computer file without patient names to eliminate any risk for medical or psychological confidentiality. Only the participating investigators, the study statistician, and two data analysts had access to the data.

### 2.4 Statistical analysis

We initially computed descriptive statistics for the baseline variables in the post-polio patient and control groups. Baseline bone biomarkers and clinical variables were compared between post-polio patients and controls using two sample t-tests for continuous variables, and Chi square test or Fisher’s exact tests for categorical variables. Changes in bone biomarkers from baseline to at least six months after starting bisphosphonate treatment were assessed using paired t-tests within each group. Differences in bone biomarker changes between the two groups were further analyzed using two-sample t-tests, and adjusted differences in bone biomarker changes between the two groups were estimated using multivariable linear regression models after adjusting for age, sex, and body mass index (BMI) at baseline. In addition, adjusted differences in bone biomarker changes between the two groups were estimated with multivariable linear regression, adjusting only for baseline biomarker values. Lastly, multivariable linear regression models were constructed to examine the relationship between baseline 25D levels (sufficient 25D > 75 nmol/L vs insufficient 25D ≤ 75 nmol/L) and changes in bone biomarkers after at least six months of bisphosphonate treatment while adjusting for polio status, and baseline age, sex, and BMI. All tests were two-sided, with p-values ≤ 0.05 considered statistically significant. Data analysis was conducted using RStudio version 2021.090 + 351 (32).

## 3 Results

Two hundred and twenty-five patient charts (170 post-polio and 55 non-polio) were reviewed for the study. After completion of the initial review, 169 patients (66%) were excluded primarily due to unavailability or inappropriate timing of bone biomarkers or BMD results. The remaining 25 post-polio patients (age 66.3 ± 8.1 years) and 31 non-polio patients (age 67.83 ± 11.3 years) with osteoporosis were further evaluated. All included post-polio patients had PPS.

The clinical characteristics and bone biomarker values of the two groups are presented in **Table 1**. Age, sex, ethnicity, and proportion of menopausal women and those with a smoking history were similar between the two groups. BMI and eGFR were higher in post-polio patients. (eGFR may be higher in the post-polio group due to lower muscle mass with consequent lower creatinine levels). With regard to chronic disease, 3 post-polio patients and 2 controls had diabetes mellitus, 3 post-polio patients and 5 controls had a history of cancer, and one control had a fatty liver. No patients in the post-polio group and one patient in the control group was treated with hormone replacement therapy. In the post-polio group, 19 patients were ambulatory, and 6 used a wheelchair for mobility. BMD measurements and T-scores were higher at the spine, but lower at the hip in post-polio patients compared to non-polio controls. Mean bone biomarker values were within normal limits in both groups. For 25D, 6 post-polio patients and 5 controls had insufficiency (25 to <75 nmol/L), and none had 25D deficiency (<25nmol/L) at baseline. However, when comparing post-polio patients with non-polio controls, post-polio patients had significantly lower PTH, non-specific ALP, OC, and CTX levels than non-polio controls. We also compared sBTM’s in females only between the two groups. ALP was similar in both groups, but OC and CTX were significantly lower in the post-polio group compared to controls (18.34 ± 5.86 vs 27.87 ± 8.61, 95% CI -16.12 to -2.94, p=0.01, and 0.28 ± 0.18 vs 0.60 ± 0.20, 95% CI -0.48 to -0.16, p<01.), All included post-polio and control patients were treated at the Bone Metabolism Clinic of a University-affiliated hospital. Patients included in the treated group (15 post-polio and 20 non-polio patients) were treated (with alendronate or risedronate) for at least six months. Treatment time from initiation of bisphosphonates to measurement of bone biomarkers ranged from a median of 509 to 597 days for post-polio patients and 486 to 564 days for the non-polio controls.

**Table 1:** Baseline clinical characteristics and bone biomarkers in post-polio patients and Non-Polio Controls.

Characteristic/Marker	Post-Polio	Non-Polio	Difference	95% CI	p-Value
	(n=25)	(n=31)			
Age (years)	66.28 ± 8.09	66.23 ± 10.94	0.05	-5.22, 5.32	0.98
Sex (n, % female)	13/25 (52%)	19/31 (61%)	-9%	-39%, 20%	0.67
Body mass index (kg/m^2^)	27.96 ± 5.89	24.75 ± 3.69	3.21	**0.48, 5.95**	**0.02**
Menopausal females (n,%)	12/13 (92%)	19/19 (100%)	0^a^	0, 26.68^a^	0.41^a^
Ethnicity (n, % Caucasian)	24/25 (96%)	26/31 (84%)	4.50^a^	0.46, 226.94^a^	0.21^a^
Smoking	10/25 (40%)	5/18 (28%)	12%	-45%, 21%	0.61
eGFR	99.67 ± 12.55	85.84 ± 14.64	13.82	**6.21, 21.43**	**<0.01**
Age at polio (years)	4.46 ± 5.63				
Time since polio (years)	63.19 ± 10.08				
Severity of acute polio (0–6 scale)	2.25 ± 0.97				
Spine BMD (g/cm^2^)	1.10 ± 0.17	0.89 ± 0.116	0.21	**0.13, 0.30**	**<0.01**
Spine T-score	-0.99 ± 1.5	-2.6 ± 1.67	1.67	**0.97, 2.36**	**<0.01**
Hip BMD (g/cm^2^)	0.62 ± 0.08	0.76 ± 0.07	-0.14	**-0.18,-0.1**	**<0.01**
Hip T-score	-3.21 ± 0.56	-2.08 ± 0.63	-1.13	**-1.46,-0.8**	**<0.01**
PTH (pmol/L)	4.40 ± 1.69	5.55 ± 2.30	-1.15	**-2.31, 0.00**	**0.05**
25D (nmol/L)	81.76 ± 27.66	87.79 ± 28.74	-6.03	-21.89, 9.82	0.45
Calcium (mmol/L)	2.39 ± 0.08	2.43 ± 0.12	-0.04	-0.10, 0.02	0.2
Alkaline phosphatase (U/L)	63.88 ± 15.76	76.25 ± 26.73	-12.38	**-24.44, -0.31**	**0.04**
Osteocalcin (ug/ml)	18.31 ± 8.83	26.95 ± 8.36	-8.64	**-13.83, -3.46**	**<0.01**
C-telopeptide (microgram/L)	0.35 ± 0.25	0.55 ± 0.21	-0.2	**-0.34, -0.06**	**0.01**

Values presented are mean ± standard deviation. 95% CI = 95% confidence interval of mean difference; eGFR, estimated glomerular filtration rate PTH. parathyroid hormone. The hip BMD and T-scores presented are for the worst hip for the post-polio patient group, and the left hip for the non-polio group. ^a^ is the estimated odds ratio (OR), 95% CI for OR, and the p-value from a Fisher’s exact test. Statistically significant values are in bold. Normal values for bone biomarkers are: PTH: 1.5-9.3 pmol/L; 25D: <25 nmol/L deficient, 25–50 nmol/L insufficient, >75 nmol/L sufficient; Calcium: 2.12-2.62 mmol/L; ALP: 18-60M: 53–128 U/L and >60M: 56–120 U/L, 18-60F: 42–98 U/L and >60F: 53-141U/L; Osteocalcin: >18F: 5–36 ng/L and >35M: 5-35ng/L; C-telopeptide: 40-50M: 0.18-0.80, 50-60M: 0.16-0.74, 60-70M: 0.13-0.75. >70M 0.12-0.78 ng/mL, 40-50F: 0.13-0.67, 50-60F: 0.18-1.06, 60-70F: 0.17-0.97, >70F:0.15-0.86, Pre-menopause: 0.14-0.69, post-menopause: 0.18-1.02 ng/mL.

We compared the changes in bone biomarkers after at least six months of bisphosphonate treatment within the two groups (**Table 2**). There was a significant reduction in calcium, non-specific ALP, OC, and CTX in the post-polio group. Similar results were observed in the non-polio control group with significant reductions in non-specific ALP, OC, and CTX with bisphosphonate treatment. We also compared bone biomarker values between the two groups after at least six months of treatment and found no significant differences between the two groups.

**Table 2:** Bone biomarkers before and with bisphosphonate treatment (at least six month of use) in post-polio patients and non-polio controls: within and between group analysis.

	Post-Polio Patients					Non-Polio Controls					Difference of Differences (Post-Polio vs Non-Polio)		
Marker	n	Baseline	Follow-up	Diff.	95% CI	n	Baseline	Follow-up	Diff.	95% CI	Diff.	95% CI	p-Value
PTH (pmol/L)	13	4.34 ± 1.83	5.56 ± 3.28	1.23	-0.15, 2.60	19	5.60 ± 2.15	6.01 ± 2.66	0.41	-1.00, 1.82	0.73	-1.16, 2.79	0.44
25 D (nmol/L)	12	80.62± 36.05	79.35 ± 22.45	-1.28	-15.06, 12.51	20	89.10± 30.17	93.20± 19.48	4.10	-7.36, 15.57	-5.37	-22.91, 12.15	0.54
Calcium (nmol/L)	14	2.40 ± 0.07	2.31 ± 0.15	-0.09	**-0.17, -0.01**	19	2.41 ± 0.12	2.38 ± 0.08	-0.02	-0.07, 0.02	-0.06	-0.15, 0.02	0.12
ALP (U/L)	15	68.20± 15.82	57.73 ± 12.80	-10.47	**-16.51, -4.42**	17	70.53± 19.76	53.00± 12.94	-17.53	**-23.59, -11.47**	7.06	-1.18, 15.30	0.09
Osteocalcin (ng/ml)	13	19.08± 10.66	14.64 ± 8.58	-4.43	**-7.57, -1.30**	16	27.61± 7.99	14.16± 4.16	-13.45	**-17.17, -9.72**	9.01	**4.22, 13.81**	**<0.01**
C-telopeptide (mcg/L)	13	0.40 ± 0.28	0.20 ± 0.14	-0.19	**-0.36, -0.02**	15	0.60 ± 0.21	0.18 ± 0.11	-0.42	**-0.52, -0.32**	0.22	**0.05, 0.41**	**0.02**

Values presented are mean ± standard deviation. Differences presented are follow-up values minus baseline. n refers to the number of pairs used in the calculation; 95% CI, 95% confidence interval of mean difference; Diff., Difference; PTH, parathyroid hormone; ALP, alkaline phosphatase. Statistically significant values are in bold.

We then compared the changes of bone biomarkers after at least six months of bisphosphonate treatment between the two groups (**Table 2**). We found that the reduction in the sBTM’s (OC and CTX) was significantly greater in the non-polio control group compared to the post-polio group. The differences in the changes between the two groups were not significant for PTH, 25D, calcium, and non-specific ALP. Using multivariable linear models, we also compared the changes in biomarkers controlling for age, sex, and BMI (**Table 3**). We again found significant differences in the changes with treatment between the two groups for OC and CTX. The changes in these two markers were significantly greater in the non-polio control group. We also compared the biomarker changes between the two groups, adjusting only for baseline biomarker values. In this analysis the change in CTX was no longer significantly different between the two groups, but the difference in OC change remained significant (p=0.01).

**Table 3:** Comparing the changes in biomarker values with at least six months of bisphosphonate treatment between post-polio patients versus non-polio controls after adjusting for baseline age, sex, and BMI.

Marker	Estimate (Post-Polio vs Non-Polio)	95% CI	p-Value
PTH (pmol/L)	0.85	(-1.79, 3.49)	0.52
25 D (nmol/L)	-10.65	(-33.06, 11.75)	0.34
Calcium (nmol/L)	-0.09	(-0.19, 0.01)	0.09
ALP. (U/L)	2.87	(-6.84, 12.58)	0.55
Osteocalcin (ng/ml)	**8.85**	**(2.22, 15.49)**	**0.01**
C-telopeptide (mcg/L)	**0.36**	**(0.12, 0.59)**	**<0.01**

Regression estimates are presented. 95% CI, 95% confidence interval of the estimated coefficient; p-Value indicates the significance of the estimated coefficient. PTH, parathyroid hormone; ALP, alkaline phosphatase. Statistically significant values are in bold.

Using linear regression models, we evaluated the relationship between baseline 25D levels (sufficient vs insufficient; > 75 vs ≤ 75 nmol/L) and changes in bone biomarker levels with bisphosphonate treatment for six months, adjusting for polio status, and baseline age, sex, and BMI. We found no significant relationships between 25D levels and change in bone biomarkers (**Table 4**). We also calculated linear regression models to evaluate the relationship of baseline 25D with changes in bone biomarkers, adjusting only for baseline marker values. In this analysis, no significant relationships were found between 25D levels and the changes in bone biomarker values.

**Table 4:** Relationship of baseline vitamin D levels (sufficient versus insufficient) and changes in bone biomarkers with at least six months of bisphosphonate treatment after adjusting for polio status and baseline age, sex, and BMI.

Marker	Estimate (Sufficient vs Insufficient)	95% CI	p-Value
PTH (pmol/L)	-0.21	(-2.72,2.31)	0.87
Calcium (nmol/L)	0.02	(-0.08, 0.13)	0.65
ALP. (U/L)	-1.75	(-11.28, 7.79)	0.71
Osteocalcin (ng/ml)	-1.32	(-7.40, 4.75)	0.66
C-telopeptide (mcg/L)	-0.04	(-0.26, 0.18)	0.73

Regression estimates are presented. 95% CI, 95% confidence interval of the estimated coefficient; PTH, parathyroid hormone; ALP, alkaline phosphatase. p-Value indicates the significance of the estimated coefficient.

## 4 Discussion

Osteoporosis is a common and important difficulty for post-polio patients. Methods to monitor bisphosphonate treatment response are needed in this patient population. In this study we are the first to present data on bone biomarkers (including bone turnover markers of formation OC and ALP, and resorption CTX) in osteoporotic post-polio patients, evaluate their response to bisphosphonate treatment, and compare the results to those in osteoporotic controls without neurological disease. We found that several bone biomarkers were significantly lower in post-polio patients compared to controls (**Table 1**). With bisphosphonate treatment, bone turnover markers declined significantly in both groups, however the reduction was significantly smaller in the post-polio patients for OC and CTX (**Table 2**). These results indicate that despite bone biomarkers being lower at baseline in post-polio patients, this population may still benefit from biomarker monitoring of bisphosphonate treatment response.

We report that baseline levels of the bone biomarkers PTH, non-specific ALP, OC, and CTX are significantly lower in osteoporotic post- polio patients than in osteoporotic control patients without neurological disease. Therefore, bone turnover appears to be lower in post-polio patients than in non-polio controls. This finding could be related to the direct effect of the disease or be secondary to prolonged inactivity and the impact of absent or reduced bone-muscle tension on bone metabolism in the post-polio patient population.

In addition, we found that the serum levels of non-specific ALP, OC, and CTX significantly decrease in both groups in response to at least six months of treatment with bisphosphonates. These findings agree with previous observations in post-menopausal women with osteoporosis, which showed that bisphosphonates highly suppress the activity of OC and CTX, and then return to equilibrium based on feedback regulation (33). Studies showed that with oral bisphosphonate treatment, there is an early decrease in bone turnover markers and a later average decrease of 17.1% in OC, whereas CTX decreases and achieves a relative balance at around two years. This pattern synergy between OC (considered to be a marker of bone formation) and CTX (marker of bone resorption) changes is expected since these processes are coupled (24, 34). Consequently, BMD increase would reach a plateau within three years in post-menopausal osteoporosis (33).

We found that in both groups, OC and CTX changes are of higher magnitude than changes in non-specific ALP. This finding is similar to that reported in studies in other patient populations. It is not known why oral bisphosphonates consistently result in a greater decrease in OC and CTX than non-specific ALP (24, 35). It is possible that testing for a bone-specific ALP isoform would have yielded greater changes, but this test is not commonly available in the clinical setting. There is a clear link between compliance with treatment and BTM change. The IMPACT study found that adherence to risedronate therapy was positively associated with bone turnover marker change (24). Considering the BMT changes in our post-polio and control groups, post-polio patients likely had similar compliance as the control group in our study.

The magnitude of the decline with bisphosphonate treatment was significantly greater in the control group than in the post-polio patients for OC and CTX. This likely reflects the lower baseline levels of the markers in the post-polio patients compared to the controls. However, the levels still decline with treatment in the post-polio patients, and the levels of these two markers were similar in the two groups at follow-up. The magnitude of decline in CTX levels was at least 25% in both groups (50% and 70%) indicating an adequate treatment response to bisphosphonate (27).

We did not find a significant relationship between baseline 25D levels and CTX response to bisphosphonate treatment. When controlling for baseline age, sex and BMI, patients with sufficient baseline 25D did not have a greater reduction in CTX with bisphosphonate treatment. We obtained similar findings when we evaluated this relationship correcting only for baseline biomarker level. This finding differs from that reported in postmenopausal osteoporotic patients (36, 37) where 25D levels were related to sBTM response to bisphosphonate use. Further study in this area is recommended.

This study has some limitations. As a retrospective study, our data was limited to that available in the patient clinic charts. In addition, the type of bisphosphonate used and dosage, and laboratory for measuring the bone biomarkers and BMD’s were not the same for all subjects. However, most of the bone biomarker and BMD assessments for both patient groups were completed at the same center, the MUHC. A small proportion of our patients had chronic diseases which could affect the diagnosis of osteoporosis. Nevertheless, the proportions were similar between the two groups. Our results may not be applicable to the general population of post-polio patients because only post-polio patients attending a University-affiliated post-polio clinic were included. Patients attending our clinic may be weaker and more disabled than the general population of post-polio patients. In addition, our study population was relatively small, and this prevented a reliable assessment of bone biomarker response to bisphosphonate treatment in males and females separately. We also did not have data on exact calcium intake, but most patients in both groups were taking calcium supplements. Despite these difficulties, this study provides valuable information for the management of osteoporosis in this patient population.

## 5 Conclusion

In this retrospective study, we found that several bone biomarkers including OC and CTX were significantly lower in post-polio patients with osteoporosis compared to non-polio controls with osteoporosis, indicating reduced bone turnover in the post-polio group. With bisphosphonate treatment, we observed a significant reduction in sBTM’s in both groups with a significantly greater reduction for OC and CTX in the non-polio controls, but similar mean values after treatment in both groups. These results indicate that sBTM’s could potentially be used in addition to bone densitometry measurement to monitor treatment response to bisphosphonates in post-polio patients. Although these results appear to show positive changes in sBTM’s in the post-polio population with bisphosphonate treatment, they cannot be used to conclusively recommend bisphosphonate treatment in the post-polio patient population due to our relatively small sample size and retrospective nature of this study. We recommend further prospective studies in this area including the evaluation of the effect of other anti-resorptive therapies such as denosumab and intravenous bisphosphonates, and anabolic therapies, with assessment of fracture history and bone remodeling evaluation.

## Data Availability

The datasets presented in this article are not readily available because this was not approved by our research ethics board and contains personal data. Requests to access the datasets should be directed to DT, daria.trojan@mcgill.ca.
